# Exploring the relationship of social networks on team effectiveness: a cross-cultural study of collegiate student-athletes in Korea and Hong Kong

**DOI:** 10.3389/fspor.2025.1639370

**Published:** 2025-09-17

**Authors:** Seungmo Kim, Sanghyun Park, Adam Love

**Affiliations:** ^1^ Department of Sports and Health Sciences, Hong Kong Baptist University, Kowloon Tong, Hong Kong, Hong Kong SAR, China; ^2^ Department of Sport for All, Korea National Open University, Seoul, Republic of Korea; ^3^ Department of Kinesiology, Recreation and Sport Studies, University of Tennessee, Knoxville, TN, United States

**Keywords:** social network, team cohesion, team effectiveness, collegiate athletic, cross-cultural

## Abstract

The purpose of the current study was to explore the relationship between college sports teams’ social networks (i.e., cohesion) and performance (i.e., effectiveness) within distinct sports cultures—specifically, elite sports in Korea vs. recreational sports in Hong Kong. A total of 600 student-athletes participated in a survey, comprising 256 athletes from 30 teams (12 men's teams and 18 women's teams) in Korea and 344 athletes from 27 teams (14 men's teams and 13 women's teams) in Hong Kong. Based on their response regarding advice network among teammates, total 57 Teams were categorized into dense (high advice network density) and sparse (low advice network density) based on median advice density values (S. Korea: 0.388, Hongkong: 0.431), resulting in four groups: 1. dense advice network in Hong Kong, 2. sparse advice network in Hong Kong, 3. dense advice network in Korea, and 4. sparse advice network in Korea. An ANCOVA analysis was conducted on the sub-dimensions of team effectiveness (i.e., effort, ability, preparation, persistence, and unity) to compare means across these groups. The study found that teams in Korea demonstrated greater effectiveness in terms of effort, ability, and preparation compared to those in Hong Kong. Interaction effect between network and nationality affected effort and utility of team effectiveness, and overall, Korea's dense network group outperformed Hong Kong's network group in terms of team effectiveness.

## Introduction

Team cohesion refers to “a dynamic process that is reflected in the tendency for a group to stick together and remain united in the pursuit of its instrumental objectives and/or for the satisfaction of member affective needs” (1). In fact, numerous studies have revealed that more cohesive teams are associated with positive group-level outcomes, such as team commitment (2), collective efficacy and improved team performance (3). For example, a cohesive basketball team is more likely to communicate effectively on the court, support each other during high-pressure moments, and persist through challenges, all of which lead to desired team outcomes.

### Social capital theory

Social capital is defined as “the sum of resources, actual or virtual, that accrue to an individual or a group by virtue of possessing a durable network of more or less institutionalized relationships of mutual acquaintance and recognition” (4). In other words, social capital refers to the advantages a person gains from having relationships within a network. According to social capital theory, the resources, information, and opportunities accessible through relationships within social networks can be fundamental drivers of both individual and collective success (5) since these networks foster norms of reciprocity, trustworthiness, and mutual support, which in turn facilitate efficient communication, cooperation, and the sharing of knowledge among members. Social capital is often conceptualized in two forms: bonding social capital, which refers to ties among individuals with similar backgrounds or interests (i.e., internal relations), and bridging social capital, which connects individuals across diverse social groups or organizational boundaries (i.e., external relations) (6). Both forms are essential—bonding social capital strengthens group cohesion and emotional support, while bridging social capital introduces new information, resources, and innovative perspectives.

Extensive research has confirmed the wide-ranging benefits of social capital, showing that it enhances individual and organizational effectiveness across numerous domains. For instance, high levels of social capital have been linked to better educational outcomes through supportive peer networks (7), improved public health via the dissemination of health information and collective action (8), greater efficacy in community governance and economic development through trust-based collaborations (9), and an increased capacity for creative problem-solving by leveraging diverse expertise and viewpoints (10). In the context of sports teams, social capital not only supports collaboration and coordination but also builds resilience, adaptability, and a shared commitment to group goals. By intentionally cultivating both strong internal relationships and broad external networks, teams and organizations can maximize the flow of resources and information, ultimately fostering a culture of sustained high performance.

### Team cohesion in sports

Because team sports involve a group of people (i.e., athletes) working together to achieve common goals (11), team cohesion serves an important role in sport performance and has been frequently examined as a key factor in developing successful sport teams (12). Given the nature of team sports, where results depend heavily on interactions among teammates, research applying a social network approach in the context of sports offers a valuable perspective. In social network analysis, network density is associated with group cohesion (13, 14) and has often been interpreted as reflecting team cohesion in sport (15, 16). Among several types of networks, advice and friendship networks have been regarded as important means of measuring network characteristics at the team level, as they can serve a key role in facilitating communication and the transmission of performance-related knowledge. For instance, Wang et al. (17) investigated advice networks to interpret the relationship between cooperative goals and team performance. Anderson and Warner (18), meanwhile, used friendship networks to measure team cohesion on a volleyball team. Further, Gibbons (19) conducted a study investigating changes in advice networks and friendship networks among teachers. Following such research, the current study adopted the concept of advice networks to assess team cohesion.

In general, a high level of density in an advice network among team members has the potential to affect knowledge and information sharing that contributes to team performance. In fact, the causal relationships between team cohesion and team performance have been explored extensively, as we further detail below.

### Relationships between team cohesion and team performance

Generally, it is well accepted that strong team cohesion can enhance overall team effectiveness because the increase in task performance that results from greater team cohesion influences team efﬁciency and goal attainment (12). In the literature, numerous studies have examined and reported a positive relationship between team cohesion and team effectiveness and performance. A meta-analysis conducted by Carron et al. (20), for example, confirmed the general relationship between team cohesion and performance in sport. Carron et al. examined the relationship between team cohesion and team performance based on type of sport (individual vs. team sports), type of team cohesion (social vs. task cohesion), gender, skill level, and age. The findings indicated a moderate, positive, and significant correlation between team cohesion and performance across various sport, cohesion type, and skill levels and age groups. The findings also indicated that the positive correlation between team cohesion and performance was significantly stronger for women compared to men. Filho et al. (21) also conducted a meta-analysis using studies from between 2000 and 2010, which found support for the general positive relationship between these two variables as well as the moderating effect of gender in the relationship.

Teach cohesion aids performance in important ways, as cohesive teams tend to demonstrate higher collective effort and motivation, driven by a sense of shared responsibility and reinforced by mutual trust and reciprocity within the group. Team cohesion is expected to also promote the sharing of expertise and resources, enhancing the group's overall ability and skills. Open communication and support among team members lead to better coordination and preparation, while strong social bonds foster persistence and resilience during challenges. Finally, effective collaboration and seamless task execution are facilitated by shared resources and information, made possible by strong social capital (22).

### Collegiate sports in Hong Kong and Korea

The current study was designed to examine cross-cultural team dynamics between Korea and Hong Kong, focusing on their distinct cultural backgrounds (e.g., collectivism vs. individualism) and sports development systems (e.g., elite-oriented vs. sport-participation). While both countries share certain Confucian cultural roots, their distinct positions within East Asian cultural frameworks provide a valuable lens to understand how national cultures influence individual and group behavior. According to Hofstede's framework (23), Korea is characterized as a strongly collectivistic society with high power distance and strong uncertainty avoidance—characteristics that theoretically support hierarchical team structures with strong internal cohesion. In contrast, Hong Kong presents a unique cultural hybrid, influenced by both Chinese collectivistic traditions and a British individualistic colonial history, resulting in moderate individualism scores and different power distance orientations compared to Korea.

In addition to cultural differences, variations in sport development systems in Hong Kong and Korea also create different environments for student-athletes, since the diverse collegiate sports environment may influence the formation of team chemistry and associated impact on team outcomes. The Korean collegiate sports system emphasizes elite development and competitive excellence, creating high-stakes environments where social capital investment becomes critical for individual and team success (2). Hong Kong's collegiate sports system, in contrast, emphasizes broader participation and recreational benefits rather than elite competition, potentially creating different incentive structures for social capital development (13). For example, the main role of collegiate sports in Hong Kong is to provide students opportunities to learn sportsmanship and encourage physical activity as an important part of the academic curriculum, while the main role of collegiate sports in Korea is to provide intensive athletic training and support to a few elite student-athletes (2). In other words, while college sport is more participatory in Hong Kong, it is focused primarily on competition in Korea.

In Hong Kong, the University Sports Federation of Hong Kong (USFHK) emphasizes collegiate sports as an integral part of academic life. In this way, the USFHK seeks to integrate sports with academics as a significant part of university culture. In the 2023–24 season, student-athletes from 13 tertiary institutions in Hong Kong participated in 18 sports. In contrast, the Korea University Sport Federation (KUSF) primarily provides more of an advisory role rather than organizing competitions, as it provides limited annual leagues for several men's sports. In fact, university competitions are mainly organized by each national sports association. While the USFHK offers the same opportunities for male and female student-athletes to participate in annual leagues, the KUSF provides only limited sports leagues for female student-athletes, which accentuates the male-dominant elite sports culture in Korea. This highlights a divergence in collegiate sports systems between Hong Kong and Korea, with Hong Kong prioritizing broad student participation and Korea focusing more on elite athlete development (2).

With this context in mind, the main goal of this study was to explore how the unique sports cultures in Korea and Hong Kong could impact the connection between team cohesion and team performance. The study's hypotheses proposed that student-athletes' attitudes and behaviors in their athletic pursuits may be influenced by the structures and characteristics of sports in each country. By examining these variations, the study aimed to uncover the relationship between culture, teamwork, and athletic success in Korea and Hong Kong. The hypotheses were as follows:

Hypothesis 1: Team effectiveness varies based on the density of the team network.

Hypothesis 2: Variations in team effectiveness are apparent based on nationality.

Hypothesis 3: The interaction effect of team network density and nationality influences team effectiveness.

## Method

### Participants and data collection

The population for this study were student-athletes participating in collegiate sports in Korea and Hong Kong. Data from Korean athletes were collected between October and December 2023, while data from Hong Kong athletes were collected between April and May 2023. The choice of these time periods was based on to the academic year disparities between two countries; Korea's academic year commences in March, whereas Hong Kong's begins in September. This scheduling ensured that participants had completed an entire season of college sport before taking the survey.

To create a survey environment where every athlete could nominate their teammates, we asked team managers to facilitate a meeting for all members of their team. Consequently, we gathered as many team members as possible and requested their participation in our survey. However, we found that some data included inaccurate responses (e.g., incorrect or inappropriate names), which were excluded from the final analysis. In addition, we excluded responses from teams where less than 50% of the members participated. Through this process, 600 data points were utilized for the final analysis. Specifically, in Korea, 256 athletes from 30 teams (12 men's teams and 18 women's teams) completed the survey, while in Hong Kong, 344 athletes from 14 men's teams and 13 women's teams participated in the survey. Consequently, the respondents engaged in 21 different sports (e.g., badminton, basketball, dragon boat, fencing, handball, rugby, table tennis, taekwondo, volleyball, water polo, shooting) across the two countries. Additional demographic information is presented in Table 1.

**Table 1:** Demographic characteristics.

Characteristics	Korea	Hongkong
Teams (participants)	30 (256)	27 (344)
Team gender (men's/women's)	12/18	14/13
Team size (average team members)	8.70	13.04
Median density of advice network	.388	.431

### Measurements

The questionnaire employed in the current study consisted of three components: a. demographic information, b. Name Generator Questionnaire (NGQ) based on social network analysis, and c. pre-existing item-based scale for team effectiveness.

The first section of the survey asked participants' demographic information (i.e., nationality, gender, and sport). We considered only three demographic variables so as to avoid survey fatigue and foster thorough, comprehensive completion of the NGQ, which requires significant cognitive effort.

The second section included the NGQ, which asked participants to nominate names of athletes in their advice network. For the NGQ component, participants were asked the following question: “Who do you ask for advice among your team members?” Participants were allowed to write five names of team members for the above question. The NGQ measurement has been utilized as a proxy of team cohesion by numerous social network studies (18, 24, 25). For instance, in Wei et al. (26) study, the relationship with team effectiveness was revealed by measuring the nurse's team cohesion in the NGQ method. In addition, Wäsche et al. (27) suggested that adoption of social network analysis (SNA) included NGQ has proven useful in sport field. SNA focuses on the relational nature of social structure rather than categories attributed to independent social units. As such, SNA represents a method to advance the substantive understanding of structures and processes constructed through or resulting in relations among social actors. NGQ, which is based on the sociometric scale rather than the indirect measurement method through a questionnaire, is effective in identifying the structural relationship of a team. In addition, criteria for identifying inappropriate responses (e.g., repetitive nomination, reference to an off-list name) were established, and these data were excluded from the final analysis.

The third section included an existing, previously validated scale for team effectiveness—20 items with five sub-factors (i.e., ability, effort, persistence, preparation, and unity) from the Collective Efficacy Questionnaire for Sports (CEQS) developed by Short et al. (28). The CEQS was used to assess student-athletes' perceptions of team effectiveness using a 7-point Likert scale ranging from 1 (strongly disagree) to 7 (strongly agree). The measurement tool was validated through a translation and back-translation process by experts. A final version was then created in two languages (Korean and Chinese) and administered to a Korean sample and a Hong Kong sample, respectively. This study received approval from the Research Ethics Committee of the Hong Kong Baptist University (REC/22-23/0372), and the informed consent was obtained from each participant prior to data collection.

### Data analysis

The current study used density of advice network to measure team cohesion. The exchange of internal advice among athletes within a sport team is critical, as the advice shared between team members can profoundly impact the team's ability to achieve its goals or desired outcomes (20). Additionally, internal advice plays a crucial role in the team's strategic decision-making process (29). While prior studies (25, 30) often used a representative value, such as the mean, of individual responses to assess team cohesion, the limitations of this indirect measurement approach have been widely recognized in the literature. In response, several researchers (18, 31, 32) have attempted to measure team cohesion more directly through density of networks, similar to the approach taken in the current study.

Advice network density measures the proportion of actual relationships among the members of a person's social network compared to the maximum number of possible relationships (33). The density calculation can be computed using the following formula.

$$\mathrm{advice}\;\mathrm{network}\;\mathrm{density}=\frac{2k}{n(n-1)}$$

In this formula, k denotes the number of advice connections present in a network, while *n* represents the total number of student-athletes in the network. Advice network density may range from 0 to 1. For instance, if all individual players in the network were completely unconnected, the structural cohesion measure would be 0. However, if all players were fully connected to each other (i.e., all members of the team are nominated by teammates as people from whom they seek advice), the structural cohesion measure would be 1. Therefore, in this study, a dense network means that members of a team are closely interconnected via advice sharing, while a sparse network would suggest the opposite. In this context, a connection refers to a relationship where two players exchange advice with each other.

Upon computing the advice density of each sports team in both countries through the provided formula, teams were categorized into two groups—dense advice network and sparse advice network—using the respective medians. In other words, the teams were divided into two groups according to being either above or below the median advice density values (.388 for Korea teams and.431 for Hong Kong teams). In turn, this process created four groups: 1. a dense advice network group in Hong Kong, 2. a sparse advice network group in Hong Kong, 3. a dense advice network group in Korea team, and 4. a sparse advice network group in Korea (D).

**Figure 1:**
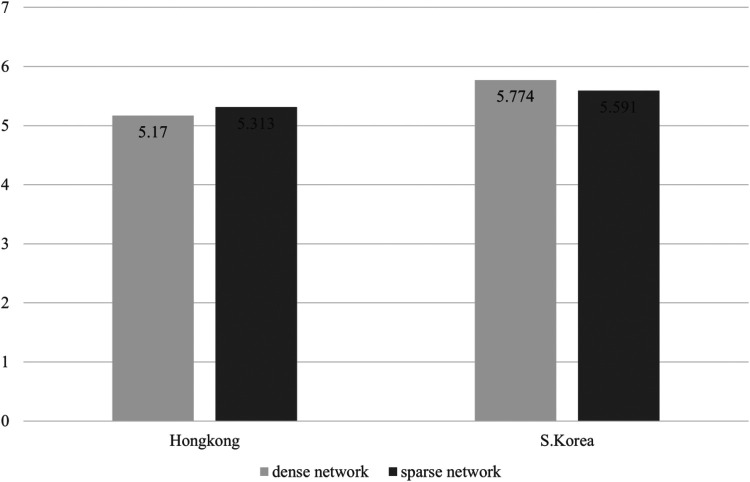
Mean difference of effort depending groups consisted of density and nationality.

**Figure 2:**
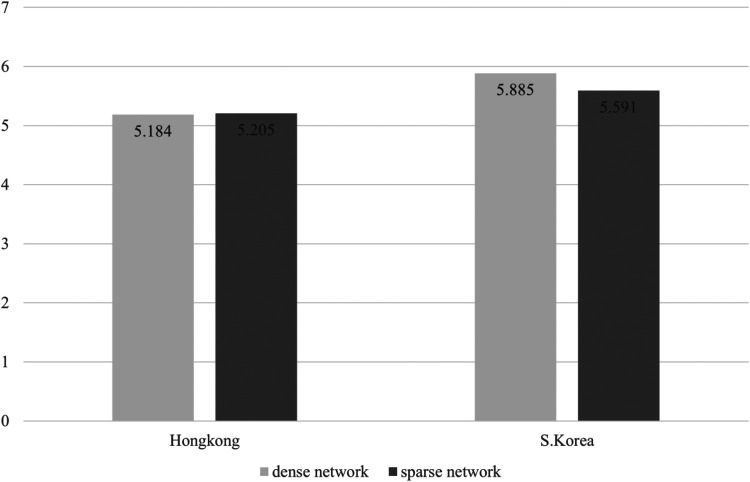
Mean difference of ability depending groups consisted of density and nationality.

**Figure 3:**
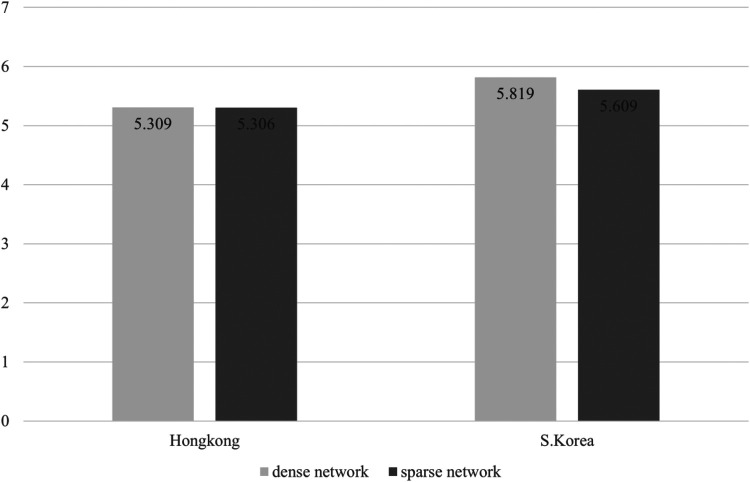
Mean difference of preparation depending groups consisted of density and nationality.

**Figure 4:**
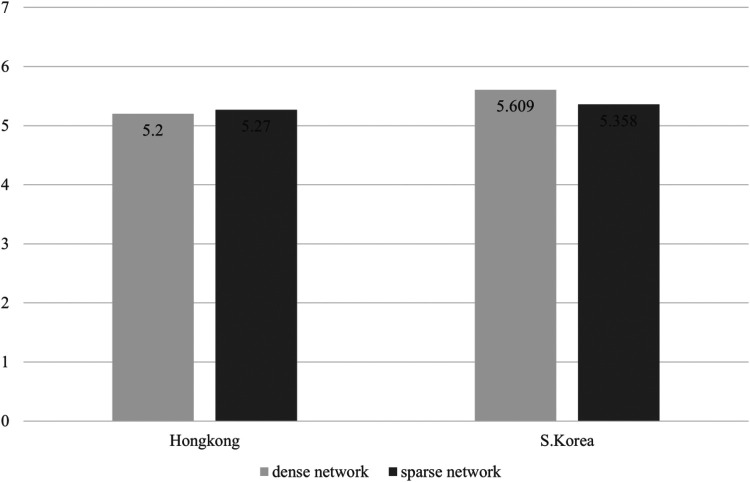
Mean difference of persistence depending groups consisted of density and nationality.

**Figure 5:**
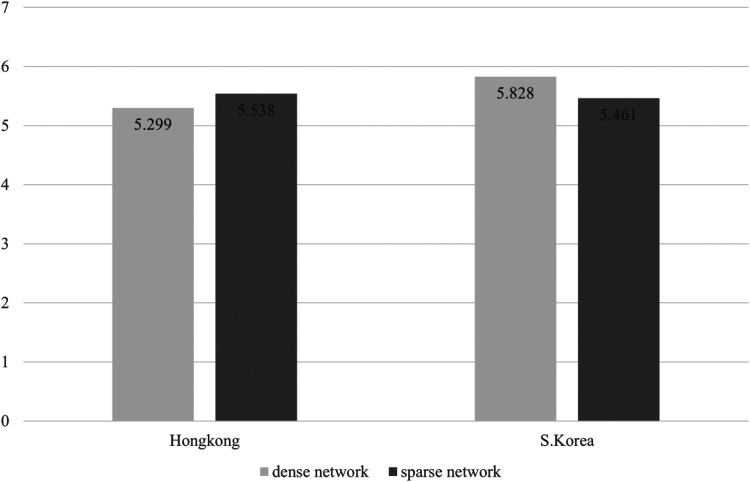
Mean difference of unity depending groups consisted of density and nationality.

Subsequently, considering that gender of team members and team size were important variables for team effectiveness, a two-way ANCOVA was conducted on sub-dimensions of team effectiveness (i.e., effort, ability, preparation, persistence, and unity). Additionally, for intuitive comparison, Figures 1–5 were displayed to compare the means across the four groups. All statistical significance levels were 0.05, and SPSS 24.0 was employed for the analysis. Also, to determine the homoscedasticity of the assumptions of ANCOVA, the Breusch-pagan test was carried out.

## Results

First, as a result of the Breusch-pagan test for equivariance, the assumption of equivariance was satisfied in all analyses (Effort: *χ*^2^ = .133, *p* = .715, Ability: *χ*^2^ = 1.620, *p* = .203, Preparation: *χ*^2^ = 1.786, *p* = .181, Persistence: *χ*^2^ = .626, *p* = .429, Utility: *χ*^2^ = .042, *p* = .828).

Second, the results indicated that network density was not a statistically significant factor for any sub-dimension of team effectiveness, which did not support Hypothesis 1. Also, nationality also did not significantly explain the variance of the sub-factors of team effectiveness, meaning Hypothesis 2 was not supported.

Third, interaction effects between network density and nationality on team effectiveness was shown to be statistically significant in Effort (*F* = 5.935, *p* = .018) and Utility (*F* = 6.620, *p* = .013), which seems to partially support Hypothesis 3. Because there is a limit to judging through statistical significance, we further analyzed the average values of sub-dimensions of the team effectiveness for each group. In this scenario, Figures 1–5 illustrated that the dense network group in Korea exhibited higher scores across the sub-dimensions of team effectiveness (e.g., effort, ability, preparation, persistence, and unity) compared to their counterparts in Hong Kong. This suggests that in situations where sports teams in Korea exhibit high cohesion, they may demonstrate greater team effectiveness than those in Hong Kong. The results of the two-way ANCOVA are presented in Table 2.

**Table 2:** Results of two-way ANCOVA**.**

Sub-dimensions	Factors	SS	df	F	p	*η* ^2^
Effort	Gender	.089	1	.370	.546	.007
	Team size	.925	1	.925	.055	.070
	Network density	.043	1	.043	.865	.035
	Nationality	2.473	1	1.787	.407	.638
	Advice density* Nationality	1.427	1	5.935	.018	.104
Ability	Gender	.491	1	1.812	.184	.034
	Team size	.511	1	1.883	.176	.036
	Network density	.008	1	.014	.920	.010
	Nationality	4.955	1	6.584	.231	.865
	Advice density* Nationality	.771	1	2.842	.098	.053
Preparation	Gender	.249	1	1.117	.296	.021
	Team size	.529	1	2.371	.130	.044
	Network density	.002	1	.005	.952	.003
	Nationality	2.977	1	7.149	.220	.873
	Advice density* Nationality	.424	1	1.900	.174	.036
Persistence	Gender	.874	1	3.316	.074	.061
	Team size	.540	1	2.050	.158	.039
	Network density	.000	1	.000	.995	.000
	Nationality	1.610	1	1.857	.399	.645
	Advice density* Nationality	.890	1	3.376	.072	.062
Utility	Gender	.163	1	.549	.462	.011
	Team size	1.011	1	3.403	.072	.053
	Network density	.117	1	.086	.813	.068
	Nationality	1.400	1	.734	.548	.421
	Advice density* Nationality	1.968	1	6.620	.013	.115

Gender and Team size are covariate.

## Discussion

The current study aimed to investigate how the distinct sports cultures in Korea and Hong Kong might influence the relationship between team cohesion and team effectiveness. We proposed three hypotheses: Hypothesis 1 suggested that team effectiveness varies based on the density of the team network; Hypothesis 2 posited that variations in team effectiveness are apparent based on nationality; and Hypothesis 3 examined whether the interaction effect of team network density and nationality influences team effectiveness. Through this investigation, we sought to understand how the structures and characteristics of sports in these two countries shape student-athletes' attitudes and behaviors in their athletic pursuits. The findings of this study provide valuable insights into the dynamics of team effectiveness in relation to network density and nationality.

First, the study's results revealed notable differences in effort, ability, and preparation levels among collegiate sports teams in Korea and Hong Kong. The dense advice network in Korea exhibited higher levels of the three dimensions in comparison to their counterparts in Hong Kong. These distinctions imply the presence of distinct cultural influences that shape the values and priorities within the respective sports environments. The emphasis on elite sport training and competition in Korea, as opposed to the more general participatory focus of Hong Kong, may explain the observed disparities in team effectiveness. The results align with the cultural values in Korea based on collectivism and an elite sport system (2, 23) that prioritizes group performance and effective group communication, which could contribute to the higher cohesion and overall effectiveness of college sports teams in South Korea. However, it is also noteworthy that no mean differences were found in persistence and unity among the groups, which suggests that while effort, ability, and preparation play crucial roles in the competitive environment of Korean college sport, other factors such as persistence and unity may not have as strong of an impact in this context.

Second, contrary to our expectations, network density or nationality was not a statistically significant factor for any sub-dimension of team effectiveness, thus not supporting Hypotheses 1 and 2. These results suggest that simply having a dense network or different nationality does not inherently lead to improved team performance. Such findings may be attributed to the presence of additional influential factors beyond structural characteristics based only on network density or nationality. For instance, team culture could significantly impact how members collaborate, share information, and resolve conflicts, potentially overshadowing the advantages of a dense network (34). In this way, structural elements like network density do not operate in isolation, and team culture may significantly influence how cohesion affects team-level outcomes. Teams characterized by a strong and supportive culture tend to exhibit higher levels of collaboration, trust, and adaptability, which can magnify the positive relationship between cohesion and performance (34). Additionally, communication styles—whether formal or informal, direct or indirect—might affect team interactions and decision-making processes, further influencing overall effectiveness (35). In addition, external influencers (e.g., organizational support and leadership styles) could also play pivotal roles in shaping team dynamics and outcomes (36) rather than network density in this study. These contextual factors may interact with network density in complex ways, suggesting that a holistic view of team effectiveness should integrate both structural and contextual elements. The unexpected findings have called for a more comprehensive study of the variables that could contribute to successful team performance, highlighting the importance of considering the interplay between network characteristics and other influential factors.

However, the interaction effect between network density and nationality on team effectiveness was supported, which confirmed our expectations, emphasizing the distinct emphasis and performance expectations present in the two countries. Korean collegiate sports prioritize elite training and competition, while Hong Kong's collegiate sport system prioritizes general participation and promoting well-being through sport (2). Therefore, the rigorous and competitive collegiate sports environment in Korea often pushes teams and athletes to prioritize winning over enjoyment, given the profound impact that success within limited opportunities as student-athletes can have on their entire athletic careers. This dynamic has led them to engage in activities that may enhance social connections (i.e., team cohesion), eventually leading to higher team effectiveness (37). In addition, collectivism in Korean society, where group harmony, collaboration, and shared achievement are highly valued (23, 38), may impact on the interaction effect since collectivist cultures often foster stronger social bonds and prioritize group success, which may explain the higher team cohesion and effectiveness observed in Korea. In contrast, Hong Kong's sports culture, based on a more individualistic orientation and a focus on general participation and well-being, may lead to less emphasis on intense group cohesion and competitive performance. In this way, our results support earlier studies which demonstrate that collectivist environments foster stronger team cohesion, more effective communication, and a greater emphasis on achieving shared objectives (39).

Recognizing the differences in emphasis between elite training in Korea and general sport participation in Hong Kong, tailored strategies should be developed to meet the specific needs and expectations of athletes in each cultural context. This may involve adjusting training methods, communication styles, and performance goals accordingly.

First, coaches should recognize that fostering open and effective communication is vital for increasing team effectiveness, rather than relying solely on team density or structural characteristics, since additional influential factors—such as effective communication—extend beyond network density or nationality. Following this recommendation, coaches should implement regular team meetings, feedback sessions, and workshops focused on building trust and understanding among team members.

Second, coaches, sport administrators, and team leaders should be mindful of the cultural influences that impact team dynamics and performance. Understanding the values and priorities of athletes from different cultural backgrounds can help foster better communication and collaboration within sports teams. For instance, coaches working in collectivist contexts such as Korea should prioritize group objectives, shared responsibility, and team-building activities, as these practices can enhance team cohesion and overall effectiveness. In contrast, coaches in more individualistic environments may benefit from placing greater emphasis on personal achievements and fostering individual autonomy, rather than focusing exclusively on team-centered accomplishments and goals.

Third, sport organizations, particularly those in highly competitive sports environments where prioritizing winning and success is paramount over enjoyment, should recognize incorporating programs that could promote effective communication and interactions. Implementing such training programs, including team-building exercises and communication workshops that emphasize teamwork and shared goals can play a critical role in enhancing team cohesion. Particularly, given the significance of effort, ability, and preparation in determining team effectiveness, coaches and sports professionals should prioritize these factors in training and development programs. Cultivating a culture of hard work, skill development, and readiness can contribute to improved team performance.

### Limitations and future research

The current study utilized advice network density as a proxy for team cohesion, which, while appropriate, may not fully capture all critical aspects of team dynamics, such as emotional support and informal social interactions that also play a role in cohesion within sports teams. Therefore, future research should incorporate additional measures that assess communication and leadership styles to gain a more comprehensive understanding of team cohesion in sports settings, including exploring the influence of emotional support and informal social interactions.

Furthermore, the findings in this study were limited to collegiate sports environments in two countries, which may limit generalizability to other organizational contexts, such as professional sports teams. To improve the broader generalizability of findings, future research should expand beyond collegiate sports environments to encompass various organizational settings, including professional sports teams. Such an approach would facilitate comparisons across different types of organizations and enhance the transferability of research outcomes to a wider array of contexts.

## Conclusions

This study highlighted two key points. First, the distinct sports cultures in Korea and Hong Kong emphasize the need for strategies tailored to the specific needs of athletes in each setting. Second, team performance is shaped by various factors beyond network density, such as team culture, communication styles, and external influences. Based on these findings, sports organizations can enhance team connections and overall performance by recognizing these cultural differences and prioritizing effective communication and teamwork.

## Data Availability

The raw data supporting the conclusions of this article will be made available by the authors, without undue reservation.
